# Comparative Outcomes Between Classic and Silent ICU Models During COVID-19 Surge in Taiwan: A Real-World Cohort Analysis from a Dual-Campus Medical Center

**DOI:** 10.3390/healthcare13233092

**Published:** 2025-11-27

**Authors:** Wei-Hung Chang, Ting-Yu Hu, Li-Kuo Kuo

**Affiliations:** 1 Department of Critical Care Medicine, MacKay Memorial Hospital, Taipei 10449, Taiwan; peacejaycool@gmail.com (W.-H.C.); lmn4093@gmail.com (L.-K.K.); 2 Department of Medicine, Mackay Medical College, New Taipei City 25245, Taiwan

**Keywords:** COVID-19, intensive care, surge capacity, ward conversion, infection control, remote monitoring

## Abstract

**Background**: During Taiwan’s May 2021 COVID-19 surge, hospitals expanded ICU capacity using both conventional and ward-converted (“silent”) ICUs. **Methods**: We retrospectively compared adults with severe COVID-19 admitted to three ICUs (two classic and one ward-based) between May and August 2021. The primary outcome was ICU mortality; secondary outcomes included ventilator-weaning success and complications. **Results**: Sixty-four patients were included (28 classic, 36 silent ICU). Baseline severity was comparable (median APACHE II 21 vs. 20). ICU mortality was 29% vs. 36% (risk difference 7.5%, 95% CI –18.6 to 33.1). No difference was found in weaning success or complication rates. No staff infections occurred. **Conclusions**: Outcomes in the ward-converted silent ICU were comparable to those in classic ICUs. With appropriate infection control and staffing, silent ICUs can safely expand surge capacity.

## 1. Introduction

ByIntroduction By early 2022, the COVID-19 pandemic had caused more than 400 million confirmed cases and 5.6 million deaths worldwide [1]. Taiwan was notably successful in containing COVID-19 during 2020, reporting only 1290 cases and 12 deaths before May 2021 [2,3]. However, a sudden large-scale outbreak centered in Taipei’s Wanhua district in mid-May 2021 led to a surge of critically ill patients, straining hospital capacities [4]. Globally, hospitals have adopted various strategies to expand intensive care unit (ICU) surge capacity, including the use of airborne infection isolation rooms (AIIRs), con-version of existing ICUs into dedicated COVID-19 units, and transformation of non-ICU areas such as operating rooms and recovery wards into functional ICUs [5,6,7,8,9,10,11]. Taiwan al-so implemented strict infection-control measures, notably the “traffic control bundle,” which had proven effective in preventing nosocomial transmission during the severe acute respiratory syndrome (SARS) outbreak and was re-applied during COVID-19 [12]. Another approach is the so-called “silent ICU,” in which general wards are rapidly converted into negative-pressure units equipped with remote-monitoring systems, ena-bling critical-care delivery while minimizing staff exposure [8,9]. Despite its conceptual advantages, the clinical outcomes of patients managed in silent ICUs compared with tra-ditional ICUs remain uncertain. In response to the May 2021 surge, MacKay Memorial Hospital (MMH), a dual-campus tertiary medical center in northern Taiwan, implement-ed all three ICU expansion models (AIIRs, converted ICU, and silent ICU). This setting provided a unique opportunity to evaluate the real-world performance of the silent ICU model. We compared patient characteristics, treatments, and outcomes between silent and classic ICUs and assessed implications for healthcare-worker safety. This study aimed to test the hypothesis that patient outcomes—including ICU mortality (primary outcome) and ventilator-weaning success (secondary outcome)—in a ward-based silent ICU would be comparable to those in conventional ICUs during a COVID-19 surge, while maintain-ing healthcare-worker safety.

## 2. Patients and Methods

### 2.1. Study Design and Setting

**Figure 1:**
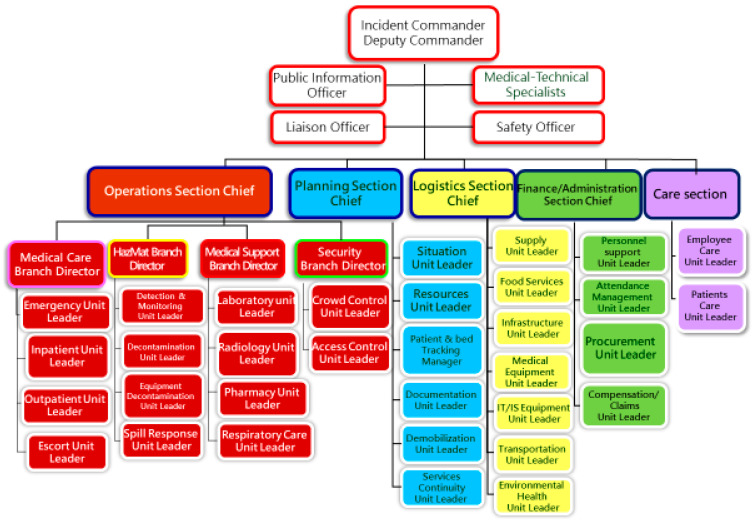
The Hospital Incident Command System (HICS) structure implemented at MacKay Memorial Hospital for coordinated COVID-19 management. The incident command framework ensured a clear chain of command and resource allocation strategy during the surge.

We conducted a retrospective cohort study at MacKay Memorial Hospital, which comprises two branches (Taipei and Tamsui) with a combined ~2000 beds. Prior to the COVID-19 surge, the hospital had 9 adult ICUs totaling 119 beds, including 4 AIIRs in the medical ICU for highly communicable diseases. A Coronavirus Pandemic Response Command Center had been in place since early 2020, operating under a Hospital Incident Command System (HICS) framework (Figure 1). In mid-May 2021, when community transmission surged, the hospital activated critical care surge plans focusing on “4S” components—Structure, Space, Staff, and Stuff—to expand ICU capacity. Within three weeks, ICU bed capacity was increased from 16 to 44 beds by repurposing existing facilities. Patients were consecutively included from all ICU admissions with laboratory-confirmed SARS-CoV-2 infection requiring invasive mechanical ventilation between 15 May and 31 August 2021.

For analytic purposes, exposure was defined as management in the ward-converted “silent ICU” (Unit C), while the non-exposure (comparison) group comprised patients admitted to conventional negative-pressure ICUs (Units A and B).

Patients were consecutively included to minimize selection bias. Allocation to each ICU model depended on bed availability rather than disease severity.

No formal sample-size or power calculation was performed due to the exploratory nature of the study.

ARDS severity was classified using the Berlin criteria, applying consistent FiO_2_ and PEEP thresholds across all ICUs.

Because Unit C (silent ICU) was opened later during the surge, potential temporal improvements in COVID-19 management were acknowledged as a limitation.

### 2.2. ICU Models and Expansion Strategy

**Figure 2:**
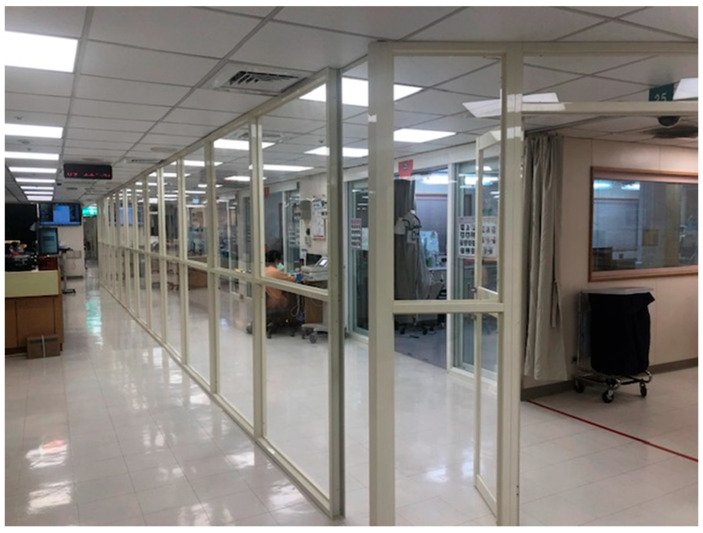
Physical partition separating the 4-bed COVID-19 zone (Unit A) from the non-COVID area in an ICU. This plastic-framed barrier and controlled workflow helped maintain separation while utilizing existing AIIR beds.

Three distinct ICUs were utilized for COVID-19 patients, representing classic and silent ICU models:

Unit A (Classic ICU—AIIRs within ICU): Unit A was a section of an existing medical ICU containing 4 negative-pressure AIIR beds. The main challenge was segregating these COVID-19 beds from other non-COVID beds in the same ICU. A floor-to-ceiling plastic partition was constructed around the AIIR area to create a sealed COVID-19 zone. Dedicated staff and one-way flow pathways (a “traffic control bundle”) were implemented to prevent cross-contamination. This measure allowed COVID-19 care to occur in Unit A while regular ICU care continued in the adjacent area with minimal risk (Figure 2). Unit A began admitting COVID-19 ICU patients on 15 May 2021.

**Figure 3:**
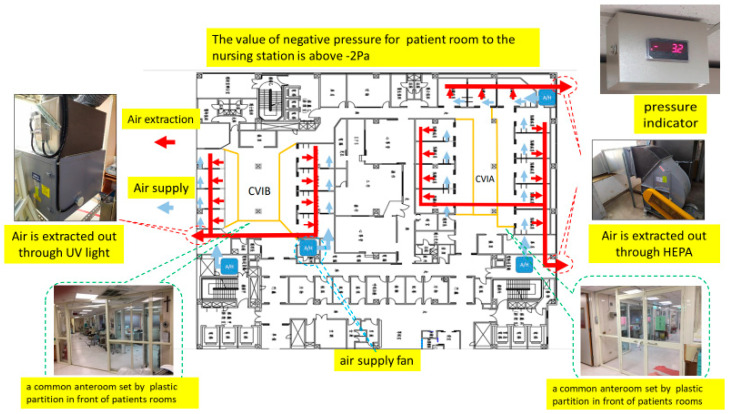
A negative-pressure COVID-19 ICU converted from a 16-bed cardiac ICU (Unit B). Industrial exhaust fans and monitors were installed to transform the space, and staff from various specialties were redeployed to this unit. CVIA = Cardiovascular ICU area A; CVIB = Cardiovascular ICU area B; Red arrows = extraction airflow; Blue arrows = supply airflow; Green dashed boxes = common anterooms. Note: Chinese characters visible on door signs in the photos indicate “Negative Pressure Isolation Room” and “No Entry”.

Unit B (Classic ICU—Converted ICU): Unit B was a 16-bed cardiovascular ICU that was entirely converted into a COVID-19 ICU. Within one week, engineering modifications were made to achieve negative pressure in this unit: three industrial exhaust fans were installed, maintaining a pressure gradient of approximately −3 to −5 Pa from the isolation rooms to the nursing station. Although these converted negative-pressure isolation rooms (NPIRs) lacked anterooms like standard AIIRs, they provided adequate ventilation and containment. All cardiac ICU patients were relocated or deferred (elective surgeries were postponed). Unit B began operating as a COVID ICU by late May 2021. Some staff in this unit initially experienced anxiety and stress due to working outside their usual specialty and concern over the improvised negative-pressure setup. Regular psychological support and training were offered (including simulation drills to reinforce infection control [13]), and confidence improved after a few weeks as staff gained experience caring for COVID-19 patients, including those requiring advanced therapies like extracorporeal membrane oxygenation (ECMO). The negative-pressure configuration and airflow layout of the converted ICU are illustrated in Figure 3.

### 2.3. Technical Specifications

Unit B’s converted ICU used three 18-inch centrifugal exhaust fans (airflow 2800–3200 m^3^/h each), maintaining a negative pressure gradient of –3 to –5 Pa relative to the corridor.

Unit C’s temporary negative-pressure system consisted of portable axial exhaust fans (2200 m^3^/h each) connected via flexible ducts to exterior windows, achieving air changes of approximately 12 per hour. Each room’s exhaust flow was monitored daily using an anemometer and pressure gauge, and HEPA filtration units were installed at the exhaust outlets.

**Figure 4:**
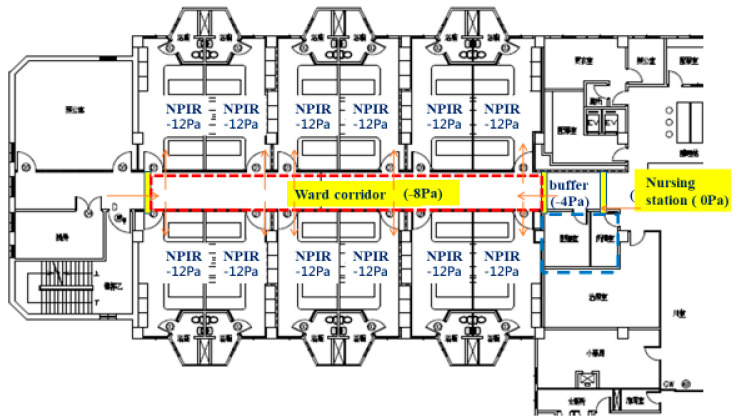
A general ward at the Tamsui branch converted into a “silent ICU” (Unit C) for COVID-19. Each patient room was retrofitted with negative-pressure capability, ICU monitoring equipment, and bedside video cameras to enable continuous remote monitoring. Note: Chinese characters in the architectural layout indicate room functions such as pharmacy storage, office, meeting room, and equipment rooms.

Unit C (Silent ICU—Converted Ward): Unit C was a general ward (approximate capacity 24–28 beds) in the Tamsui branch that was rapidly transformed into an ICU, dubbed a “silent ICU.” Construction teams installed temporary negative-pressure systems to convert each patient room into a negative-pressure chamber. Essential ICU infrastructure such as medical gas outlets and suction lines were ensured for each bed. Each room was equipped with a high-grade physiologic monitor (with central monitoring at the nurses’ station), a ventilator, infusion pumps, and a portable video camera with zoom and night-vision functions. This allowed continuous remote observation of patients from outside the isolation rooms, minimizing the frequency of staff entry. An experienced critical care physician was designated as team leader for Unit C, and a few veteran ICU nurses were redeployed to work alongside ward nurses. These measures enabled Unit C to function as a fully capable ICU with a leaner physical presence of staff (hence “silent”). Unit C opened in early June 2021 once patient numbers exceeded the capacity of Units A and B. It primarily managed patients with severe COVID-19 pneumonia requiring mechanical ventilation and routine critical care (prone positioning, titration of ventilator settings, etc.), while patients needing highly specialized interventions (e.g., ECMO) could be transferred to Unit B if necessary. The physical arrangement and monitoring system of the silent ICU are shown in Figure 4.

**Figure 5:**
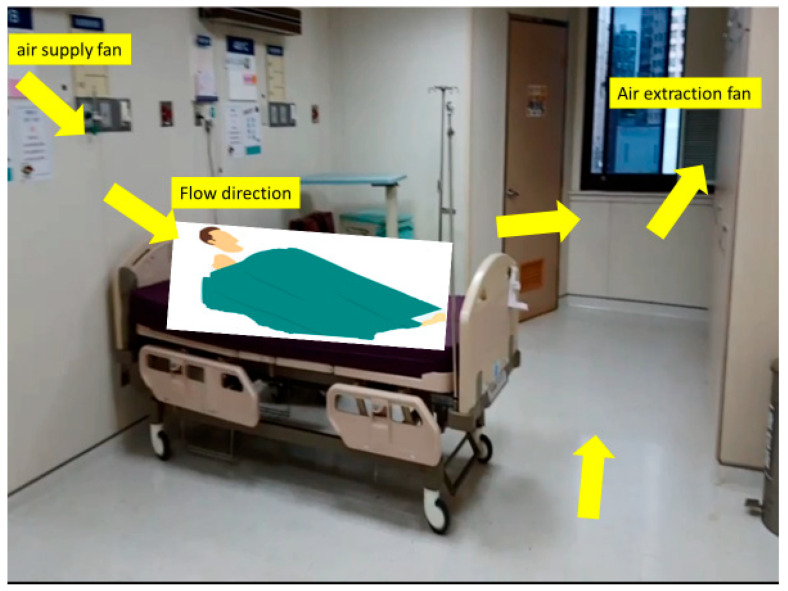
Schematic of airflow in the silent ICU (Unit C). Airflow (yellow arrows) indicates direction from corridor to patient rooms, creating negative pressure that confines contaminated air and prevents it from escaping into clean areas.

Figure 5 schematically presents the airflow direction and negative-pressure mechanism in the silent ICU.

All three COVID-19 ICUs were operated under strict infection control protocols. Staff in each unit worked in dedicated teams that did not cross over to other areas, to avoid any potential contagion spread. Full personal protective equipment (PPE)—including N95 respirators, face shields, gowns, and gloves—was mandatory for all patient contacts. Donning and doffing procedures were monitored by infection control personnel. Each unit’s layout provided a clear demarcation of clean vs. contaminated zones, with anteroom or buffer areas used in Units A and C for entering/exiting the COVID areas. Equipment and consumables were kept separate. Additionally, routine environmental disinfection was conducted, and healthcare workers underwent regular SARS-CoV-2 PCR screening. For the purpose of this study, exposure was defined as management in the ward-converted silent ICU (Unit C), while the non-exposure (comparison) group comprised patients admitted to classic ICUs (Units A and B). This classification reflects the structural and operational differences between the ward-based remote-monitoring ICU and conventional negative-pressure ICUs.

### 2.4. Data Collection

We reviewed electronic medical records, ICU logs, and the hospital COVID-19 registry to obtain data on all patients admitted to these three COVID ICUs from 15 May to 31 August 2021. Collected variables included patient demographics (age, sex), comorbidities (based on medical history and Charlson Comorbidity Index components), and acute illness severity on ICU admission. Severity was assessed by the Acute Physiology and Chronic Health Evaluation II (APACHE II) score, calculated from data within 24 h of ICU admission. We recorded key treatments and interventions, such as use of antiviral therapy (remdesivir), corticosteroids, immunomodulators (e.g., tocilizumab), prophylactic anticoagulation (enoxaparin), prone positioning, non-invasive ventilation prior to intubation, renal replacement therapy, and tracheostomy. Outcomes of interest were ICU mortality, defined as death during the ICU stay, and ventilator-weaning success, defined as successful liberation from mechanical ventilation (extubation) followed by survival to ICU discharge. Patients who remained ventilator-dependent but survived to be transferred out were categorized separately. Additional outcomes included ICU length of stay (LOS) and complication incidences: we specifically tracked pneumothorax (barotrauma), ventilator-associated pneumonia (VAP), bloodstream infections/sepsis, and acute kidney injury (AKI, including those requiring dialysis). Finally, to evaluate healthcare worker safety, we documented any COVID-19 infections among ICU staff (confirmed by routine surveillance and symptom-triggered PCR testing).

### 2.5. Statistical Analysis

Patients were grouped by ICU model (classic ICU group combining Units A and B vs. silent ICU group Unit C) for outcome comparison. Continuous variables are presented as mean ± standard deviation (SD) or median with interquartile range (IQR), as appropriate to their distribution. Categorical variables are presented as counts and percentages. We used the chi-square test or Fisher’s exact test for comparing categorical outcomes (e.g., mortality, weaning rates, complications) between groups, and Student’s *t*-test or the Mann–Whitney U test for continuous variables (e.g., age, LOS, APACHE II score). A two-sided *p*-value < 0.05 was considered statistically significant. All analyses were performed using standard statistical software (IBM SPSS Statistics for Windows, Version 26.0; IBM Corp., Armonk, NY, USA). Given the observational nature of the study, no imputation was made for missing data. Continuous variables were assessed for normality, and non-normally distributed data were reported as median (IQR). This study was reviewed and approved by the MMH Institutional Review Board (IRB No. 22MMHIS042e), which waived the requirement for informed consent due to the retrospective design and use of de-identified data. Given the small sample size (<30 per group), non-parametric tests (Mann–Whitney U and Fisher’s exact tests) were preferentially used for group comparisons to ensure conservative estimation. Given the small cohort size, no * a priori * sample-size or power calculation was performed. The analyses were exploratory and hypothesis-generating. Non-parametric tests (Mann–Whitney U and Fisher’s exact) were preferentially applied for conservative estimation. Allocation to each ICU model was determined by bed availability rather than clinical severity; baseline comparability was assessed to mitigate selection bias.

### 2.6. Ethical Considerations

This study was approved by the Institutional Review Board of MacKay Memorial Hospital (IRB No. 22MMHIS042e). The requirement for informed consent was waived due to the retrospective design and anonymized data.

## 3. Results

### 3.1. ICU Expansion and Patient Distribution

The ICU surge expansion was accomplished within three weeks of the outbreak, increasing MMH’s negative-pressure ICU capacity from 16 to 44 beds. Unit A (AIIRs in medical ICU) operated from 15 May through August 2021 with 4 functional beds; Unit B (converted CVICU) from late May through August with up to 16 beds; and Unit C (silent ICU in ward) from early June through September 2021 (gradually scaling up to ~24 beds capacity as needed). In total, 64 patients with critical COVID-19 were admitted to these units during the study period. Of these, 28 patients were treated in classic ICUs (13 in Unit A and 15 in Unit B) and 36 patients were treated in the silent ICU (Unit C). Table 1 summarizes the baseline characteristics of the classic vs. silent ICU patient groups.

Most patients were elderly males (mean age ~68 years) with multiple comorbidities, mainly hypertension and diabetes. Baseline severity was comparable between groups (median APACHE II 21 vs. 20). The only significant difference was a higher proportion of inter-hospital transfers in the classic ICU group (36% vs. 6%, *p* = 0.003) (Table 1).

### 3.2. Treatments and Interventions

All patients received dexamethasone and guideline-directed care for severe COVID-19. The use of remdesivir, tocilizumab, anticoagulation, prone positioning, renal replacement therapy, and tracheostomy were similar between groups (all *p* > 0.05). Advanced respiratory support and adjunctive therapies were provided equivalently in both classic and silent ICUs, as summarized in Table 2.

All patients in both groups received similar bundles of critical care. Notably, the application of prone ventilation and rescue treatments did not differ between the classic and silent ICUs, indicating that the ward-converted ICU was able to support advanced ARDS therapies equivalently. There was also no significant delay or difference in initiating dialysis for those who needed it in the silent ICU; portable dialysis machines and trained staff were available in Unit C. The average time from intubation to first prone session and to tracheostomy was comparable between units. These findings suggest that the level of care and interventions provided in the silent ICU matched that of the traditional ICUs despite the differing physical environment.

### 3.3. Patient Outcomes

ICU mortality was 33% overall, with no significant difference between classic and silent ICUs (29% vs. 36%, *p* = 0.53). Ventilator-weaning success and other clinical outcomes were also comparable (Table 3). Median ICU and hospital lengths of stay, as well as 28-day and in-hospital mortality, did not differ significantly between groups. These results indicate that survival and recovery prospects for critically ill COVID-19 patients were equivalent in both classic and ward-converted ICUs.

There was no statistically significant difference in ICU length of stay between the groups. The median ICU LOS was approximately 2.5 weeks in both classic and silent ICUs (19 vs. 16 days, *p* = 0.97), though individual variability was large. Notably, the longest ICU stay in the study (155 days) occurred in a Unit C patient who had protracted critical illness; this outlier did not skew the median but did raise the mean LOS in the silent ICU group (silent ICU mean LOS 37 days vs. classic ICU 22 days). Excluding that outlier, the mean ICU LOS in Unit C was 25.4 days, similar to Unit B’s 24.1 days. The hospital length of stay (from admission to discharge or death) likewise showed no difference, with medians of ~25–28 days in both groups.

Time-to-event outcomes such as 28-day mortality also did not differ significantly (approximately 26–28% in each group). Internal review of survival curves showed substantial overlap between classic and silent ICU patients. These data indicate that the survival and recovery prospects for critically ill COVID-19 patients were equivalent whether they were treated in the improvised ward ICU or in standard ICUs.

### 3.4. Complications

Major complications were common, reflecting the severity of COVID-19 ARDS. As shown in Table 4, the incidences of acute kidney injury (78%), septic shock (48%), ventilator-associated pneumonia (25%), and pneumothorax (19%) were similar between groups (all *p* > 0.05). Nearly all patients experienced at least one complication, yet the comparable rates indicate that the silent ICU maintained monitoring and care quality equivalent to conventional ICUs.

### 3.5. Healthcare Worker Safety and Infection Control

No healthcare workers in any of the three COVID-19 ICUs acquired SARS-CoV-2 infection during the study period. As summarized in Table 5, there were no instances of staff exposure, PPE breaches, or environmental contamination across all units. Compliance with PPE use and hand hygiene was verified through direct observation by infection-control nurses twice weekly, supplemented by daily self-checklists signed by shift leaders. Audit results were reported to the hospital’s Infection Control Committee, with corrective training conducted immediately upon any deviation. These findings confirm that, with adequate training and strict infection control measures, the ward-based silent ICU protected staff as effectively as conventional ICUs.

## 4. Discussion

This discussion summarizes the major outcomes—including mortality, ventilator liberation, complications, and infection-control performance—across ICU models. In this real-world cohort during a COVID-19 surge, a ward-converted “silent ICU”—a general ward rapidly transformed into an ICU with remote-monitoring capability—achieved patient outcomes comparable to those of traditional ICUs. ICU mortality and ventilator-weaning success rates in the silent ICU were statistically indistinguishable from those in the two classic COVID-19 ICUs. Complication rates such as pneumothorax, ventilator-associated pneumonia (VAP), and septic shock were also similar across models. Importantly, no healthcare-associated SARS-CoV-2 infections occurred among ICU staff, demonstrating that infection control was not compromised in the repurposed unit. To our knowledge, this is one of the first studies to directly compare clinical outcomes between a “silent ICU” and conventional ICUs during a pandemic surge.

Our findings support the feasibility and safety of the silent ICU concept. Prior pandemic planning literature has discussed converting non-ICU spaces into ICUs to expand surge capacity [5,6,7,8,9]. Comparable findings were noted in Italy (Carenzo et al., 2020) and Saudi Arabia (Arabi et al., 2021), where rapid ICU expansion during COVID-19 surges maintained similar mortality rates to standard ICUs [8,9].

Concerns have been raised that such ad hoc ICUs might deliver lower-quality care because of unfamiliar environments, equipment limitations, or fewer experienced staff on-site. Concerns have been raised that such ad hoc ICUs might deliver lower-quality care because of unfamiliar environments, equipment limitations, or fewer experienced staff on-site. In our experience, these risks were mitigated through careful preparation and staffing adjustments. The silent ICU was fully equipped with standard ICU infrastructure (monitors, ventilators, infusion pumps, suction, and blood-gas analyzers) and a reliable oxygen supply, making it functionally comparable to a conventional ICU bed for patients with primarily single-organ respiratory failure. Each shift included a mix of redeployed ICU nurses and ward nurses, maintaining nurse-to-patient ratios of 1:1 or 1:2 similar to classic ICUs. A dedicated intensivist led the team, with residents supervising up to three patients each. These measures likely contributed to the comparable outcomes observed. Advanced interventions—including prone positioning and dialysis—were executed in the silent ICU without delay, confirming that a well-organized ward ICU can uphold critical-care standards.

Patient selection bias was minimal. Baseline severity (APACHE II scores, ARDS classification) was similar, indicating that silent ICU patients were not inherently less ill. Only the most resource-intensive therapy, extracorporeal membrane oxygenation (ECMO), was administered in Unit B (the converted cardiovascular ICU) because of its pre-existing ECMO capability. If larger numbers of patients had required ECMO or other complex multi-organ support (e.g., intra-aortic balloon pump or combined CRRT + ECMO), a ward ICU might have faced greater challenges. Nonetheless, even patients with shock and severe acute kidney injury were effectively managed in the silent ICU using on-site dialysis and vasopressors. Arabi et al. suggested that future critical care may increasingly rely on flexible ICU extensions, such as “pop-up” ICUs in wards [8], and our results empirically support this strategy. Early in the pandemic, experts in Asia likewise emphasized adaptive ICU designs to manage surges [14].

The outcomes we observed align with other reports. Compared with prior international reports, our observed mortality (33%) aligns closely with the 25–40% range reported by Grasselli et al. and Bhatraju et al. [15,16], supporting external consistency. However, our data uniquely add infection-control metrics rarely described in earlier surge-capacity studies. Carenzo et al. described rapid ICU expansion in Italy during 2020, converting operating rooms and wards and reporting an overall ICU mortality of 34%, similar to our 33% [9]. However, few studies have compared outcomes by ICU type. Our data add evidence that ward-converted ICUs can achieve results on par with standard ICUs. Early global reports also documented ICU mortality between 25 and 40% during initial COVID-19 waves [15,16,17]. A critical factor in our success was protection of healthcare workers: nosocomial staff infections can cripple surge capacity [18]. Taiwan’s SARS-era experience demonstrated the effectiveness of strict infection-control bundles and staff cohorting [12]. During our surge, adherence to these principles (universal PPE, controlled entry/exit, cohort staffing) resulted in zero HCW infections [19]. Even in the silent ICU, where some personnel were new to critical-care infection protocols, continuous training and in situ simulations [13] maintained compliance. Initial anxiety and stress, particularly among Unit B staff, mirrored findings from other centers [20,21]. Ongoing communication, psychological support, and demonstration of effective infection control helped restore confidence. By the end of the surge, morale was high, and staff expressed pride in providing safe care. Such support likely prevented burnout and moral injury, both recognized risks among ICU professionals during the pandemic [22,23,24,25].

Compared with prior international reports, our observed mortality (33%) aligns closely with the 25–40% range reported by Grasselli et al. and Bhatraju et al. [15,16], supporting external consistency. However, our data uniquely add infection-control metrics, rarely described in earlier surge-capacity studies.

This study offers several practical implications. First, preparedness plans should incorporate the capacity to establish temporary ICUs swiftly. In our case, a 24-bed silent ICU was created in under two weeks with minimal modifications (exhaust fans, central monitoring) and no permanent construction, demonstrating flexibility. Second, staffing such units requires creative resource mobilization. Pairing seasoned ICU nurses with ward nurses ensured knowledge transfer while maintaining standard ratios. Cross-training programs should be institutionalized so that non-ICU clinicians can be rapidly upskilled during crises. Ward nurses in Unit C became proficient in core ICU skills (e.g., ventilator checks, arterial-line care) within a week of hands-on supervision, which is promising for future surges. Similar models of mobilizing generalist staff have been described elsewhere [26,27]. Third, telemedicine and remote monitoring can amplify ICU capacity. In the silent ICU, bedside cameras and intercoms allowed continuous observation and communication, reducing unnecessary room entries, PPE use, and exposure. This approach aligns with the concept of a “virtual ICU panel,” where centralized monitoring enables fewer staff to oversee multiple patients [28,29]. Our experience mirrors broader tele-ICU initiatives that improve critical-care delivery across hospitals and address intensivist shortages [23,24,30]. These technologies may also enhance efficiency and infection control beyond the pandemic.

Despite its strengths, this study has limitations. The sample size (*n* = 64) is modest and reflects a single-center experience, limiting generalizability. Our outbreak lasted only a few months; results might differ in prolonged surges or in resource-limited hospitals. Patients were not randomized to ICU type; bed availability and logistics determined allocation. However, comparable baseline severity and standardized protocols mitigate major bias. Our silent ICU benefited from tertiary-center resources (e.g., immediate consulting and equipment access). In settings with fewer resources, ward-to-ICU conversion may pose greater challenges. Finally, we did not quantify process metrics (e.g., alarm-response times, nursing-interaction frequency), which could differ subtly between units. Future studies should incorporate such metrics and qualitative assessments of staff and patient experiences to refine silent ICU implementation.

Given the limited sample size, the findings should be interpreted as hypothesis-generating rather than confirmatory.

Although statistical equivalence could not be established because formal non-inferiority testing was not performed, the observed outcome similarity suggests potential comparability between ICU models. Future multicenter or randomized studies with predefined equivalence margins are warranted to confirm these findings and assess long-term outcomes, cost-effectiveness, and patient-centered metrics. Future research should also explore operational efficiency indicators, such as nurse workload, alarm response time, and psychological impact on redeployed staff.

Potential confounding due to temporal improvements in COVID-19 management cannot be excluded, as Unit C (silent ICU) began operation later in the surge. Further research should include multicenter cohorts, standardized case-mix adjustment, and longer-term follow-up to validate safety, cost-effectiveness, and staff well-being under ward-based ICU deployment.

Future research should also explore operational efficiency indicators, such as nurse workload, alarm response time, and psychological impact on redeployed staff.

## 5. Conclusions

The silent ICU achieved outcomes comparable to conventional ICUs, suggesting that ward-to-ICU conversion may serve as a feasible and safe surge-capacity strategy. Hospitals could consider integrating silent ICU activation into pandemic preparedness protocols, but larger multicenter studies are warranted to validate its generalizability and cost-effectiveness across diverse healthcare systems.

## Figures and Tables

**Table 1:** Baseline Characteristics of Patients in Classic ICU vs. Silent ICU.

Characteristic	Classic ICU (Units A + B, *n* = 28)	Silent ICU (Unit C, *n* = 36)	*p*-Value
Age, years (mean ± SD)	67.0 ± 13.1	68.5 ± 15.2	0.68
Male sex (%)	22 (78.6%)	30 (83.3%)	0.62
APACHE II score (median [IQR])	21 [17–27]	20 [15–24]	0.75
Any comorbidity (%)	24 (85.7%)	30 (83.3%)	0.78
Chronic hypertension (%)	18 (64.3%)	20 (55.6%)	0.47
Diabetes mellitus (%)	10 (35.7%)	12 (33.3%)	0.84
Coronary artery disease (%)	3 (10.7%)	2 (5.6%)	0.65
Heart failure (%)	5 (17.9%)	4 (11.1%)	0.49
Chronic lung disease (COPD) (%)	4 (14.3%)	5 (13.9%)	0.96
Chronic kidney disease (%)	2 (7.1%)	3 (8.3%)	1
Malignancy (%)	1 (3.6%)	1 (2.8%)	1
Severity of illness upon ICU admission			
Moderate ARDS (%)	8 (28.6%)	13 (36.1%)	0.51
Severe ARDS (%)	20 (71.4%)	23 (63.9%)	0.51
Referred from outside hospital (%)	10 (35.7%)	2 (5.6%)	**0.003**

Continuous variables presented as median (IQR) given small sample size. Abbreviations: SD—standard deviation; IQR—interquartile range; COPD—chronic obstructive pulmonary disease; ARDS—acute respiratory distress syndrome. Moderate and Severe ARDS were defined by the PaO_2_/FiO_2_ ratio on admission (Berlin criteria). *p* < 0.05 is shown in bold. Continuous variables were compared test using Student’s *t*-test or Mann–Whitney *U* test, as appropriate. Categorical variables were compared using Chi-square or Fisher’s exact depending on expected frequencies.

**Table 2:** Summary of treatments and interventions applied in classic and silent ICUs.

Treatment/Intervention	Classic ICU (A + B) (*n* = 28)	Silent ICU (C) (*n* = 36)	*p*-Value
Corticosteroid therapy (dexamethasone)	28 (100%)	36 (100%)	–
Remdesivir antiviral therapy	21 (75.0%)	27 (75.0%)	1
IL-6 inhibitor (tocilizumab)	6 (21.4%)	9 (25.0%)	0.77
Therapeutic anticoagulation instituted	27 (96.4%)	34 (94.4%)	1
Prone positioning applied	22 (78.6%)	28 (77.8%)	0.93
Non-invasive ventilation pre-intubation	5 (17.9%)	8 (22.2%)	0.76
Mean duration of MV (days) for survivors	16.5 ± 9.8	17.4 ± 10.5	0.78
Renal replacement therapy (CVVH/IHD)	8 (28.6%)	7 (19.4%)	0.39
Tracheostomy performed	2 (7.1%)	4 (11.1%)	0.68

Abbreviations: MV—mechanical ventilation; CVVH—continuous venovenous hemofiltration; IHD—intermittent hemodialysis.

**Table 3:** Clinical Outcomes. Between-group risk differences and 95% confidence intervals were calculated for key outcomes: ICU mortality difference: 7.5% (95% CI, −18.6% to 33.1%); Ventilator-weaning success difference: 13.9% (95% CI, −10.8% to 38.5%), indicating no statistically significant effect. Relative risks with 95% confidence intervals were calculated for major outcomes (e.g., ICU mortality RR 1.26 [95% CI 0.62–2.58]). Continuous variables are presented as medians (IQR) due to non-normal distributions.

Outcome	Classic ICU (A + B)	Silent ICU (C)	*p*-Value
ICU mortality rate	8/28 (28.6%)	13/36 (36.1%)	0.53
Ventilator-weaning success rate	14/28 (50.0%)	23/36 (63.9%)	0.23
Ventilator-dependent at transfer (%)	2/28 (7.1%)	4/36 (11.1%)	0.68
ICU length of stay—median (IQR), days	19 (12–27)	16 (9–31)	0.97
Hospital length of stay—median, days	28 (18–46)	25 (17–45)	0.88
28-day mortality (%)	7 (25.0%)	10 (27.8%)	0.8
In-hospital mortality (%)	9 (32.1%)	14 (38.9%)	0.57

**Table 4:** Major Complications During ICU Stay.

Complication	Classic ICU (A + B) (*n* = 28)	Silent ICU (C) (*n* = 36)	*p*-Value
Acute kidney injury (AKI)	23 (82.1%)	27 (75.0%)	0.54
AKI requiring dialysis	8 (28.6%)	7 (19.4%)	0.39
Septic shock (vasopressors required)	13 (46.4%)	18 (50.0%)	0.8
Ventilator-associated pneumonia (VAP)	7 (25.0%)	9 (25.0%)	1
Pneumothorax (barotrauma)	5 (17.9%)	7 (19.4%)	0.87
Bacteremia (non-pulmonary source)	3 (10.7%)	2 (5.6%)	0.65
Deep vein thrombosis	1 (3.6%)	0 (0%)	0.45
Myocarditis/Pericarditis	0	0	–
Any complication above	26 (92.9%)	33 (91.7%)	1

**Table 5:** Healthcare Worker (HCW) Safety and Infection Control Outcomes.

Indicator	Unit A (AIIR ICU)	Unit B (Converted ICU)	Unit C (Silent ICU)
HCW COVID-19 infections, *n*	0	0	0
HCWs quarantined due to exposure, *n*	0	0	0
Breaches in PPE protocol, *n*	0 (minor breaches promptly corrected)	0	0
Environmental contamination events, *n*	0	0	0

Note: Periodic surveillance cultures detected no significant surface or air contamination outside patient isolation rooms in any unit.

## Data Availability

The data presented in this study are available on request from the corresponding author due to patient confidentiality and institutional regulations.
